# Establishment and Validation of a Non-Invasive Diagnostic Nomogram to Identify Spontaneous Bacterial Peritonitis in Patients With Decompensated Cirrhosis

**DOI:** 10.3389/fmed.2021.797363

**Published:** 2022-01-31

**Authors:** Shoushu Xiang, Juntao Tan, Chao Tan, Qian Xu, Yuanjiu Wen, Tiantian Wang, Chen Yang, Wenlong Zhao

**Affiliations:** ^1^College of Medical Informatics, Chongqing Medical University, Chongqing, China; ^2^Department of Medical Administration, People's Hospital of Chongqing Banan District, Chongqing, China; ^3^Cancer Hospital, Chongqing University, Chongqing, China; ^4^Department of General Medicine, Army Medical University (Third Military Medical University), Chongqing, China; ^5^Medical Data Science Academy, Chongqing Medical University, Chongqing, China

**Keywords:** decompensated cirrhosis, spontaneous bacterial peritonitis, nomogram, diagnostic, non-invasive

## Abstract

**Background:**

Spontaneous bacterial peritonitis (SBP) is a common and life-threatening infection in patients with decompensated cirrhosis (DC), and it is accompanied with high mortality and morbidity. However, early diagnosis of spontaneous bacterial peritonitis (SBP) is not possible because of the lack of typical symptoms or the low patient compliance and positivity rate of the ascites puncture test. We aimed to establish and validate a non-invasive diagnostic nomogram to identify SBP in patients with DC.

**Method:**

Data were collected from 4,607 patients with DC from July 2015 to December 2019 in two tertiary hospitals in Chongqing, China (A and B). Patients with DC were divided into the SBP group (995 cases) and the non-SBP group (3,612 cases) depending on whether the patients had SBP during hospitalization. About 70% (2,685 cases) of patients in hospital A were randomly selected as the traindata, and the remaining 30% (1,152 cases) were used as the internal validation set. Patients in hospital B (770 cases) were used as the external validation set. The univariate analysis and least absolute shrinkage and selection operator (LASSO) regression were used to screen variables, and logistic regression was used to determine independent predictors to construct a nomogram to identify patients with SBP. Area under curve (AUC), calibration curve, and dynamic component analysis (DCA) were carried out to determine the effectiveness of the nomogram.

**Result:**

The nomogram was composed of seven variables, namely, mean red blood cell hemoglobin concentration (odds ratio [*OR*] = 1.010, 95% *CI*: 1.004–1.016), prothrombin time (*OR* = 1.038, 95% *CI*: 1.015–1.063), lymphocyte percentage (*OR* = 0.955, 95% *CI*: 0.943–0.967), prealbumin (*OR* = 0.990, 95% *CI*: 0.987–0.993), total bilirubin (*OR* = 1.003 95% *CI*: 1.002–1.004), abnormal C-reactive protein (CRP) level (*OR* = 1.395, 95% *CI*: 1.107–1.755), and abnormal procalcitonin levels (*OR* = 1.975 95% *CI*: 1.522–2.556). Good discrimination of the model was observed in the internal and external validation sets (AUC = 0.800 and 0.745, respectively). The calibration curve result indicated that the nomogram was well-calibrated. The DCA curve of the nomogram presented good clinical application ability.

**Conclusion:**

This study identified the independent risk factors of SBP in patients with DC and used them to construct a nomogram, which may provide clinical reference information for the diagnosis of SBP in patients with DC.

## Introduction

Liver cirrhosis (LC) results from different mechanisms of liver injury that lead to necroinflammation and fibrogenesis. Histologically, it is characterized by diffuse nodular regeneration surrounded by dense fibrotic septa with subsequent parenchymal extinction and collapse of liver structures, together causing pronounced distortion of hepatic vascular architecture (1–3). LC is responsible for increased morbidity and mortality in developed countries; it is the 14th most common cause of death in adults worldwide but the fourth in Central Europe. It causes 1.03 million deaths worldwide every year(4). About 1,70,000 individuals die from LC per year in Europe (5), whereas 33,539 die from LC annually in the United States (6). LC is divided into compensated asymptomatic cirrhosis and decompensated cirrhosis (DC). The transition from compensated asymptomatic cirrhosis to DC occurs at a rate of about 5–7% per year (7). DC is a systemic disease with multiple organ or system dysfunction (8). Patients with DC are prone to hypersplenism and decreased immune function, and they lack resistance to endogenous and exogenous bacteria. Thus, patients with DC are prone to infection of various parts and tissues.

A particular threat for patients with DC is the development of bacterial infections (9). Patients with DC may suffer from significant alterations in various parts of the immune system, which can cause complex cirrhosis-associated immune dysfunction (10). As a result, these patients have a higher susceptibility for infections but show a hyperinflammatory response as soon as an infection has been acquired. The most frequent type of infection in patients with DC is spontaneous bacterial peritonitis (SBP) (11, 12).

Spontaneous bacterial peritonitis is acute bacterial peritonitis caused by non-abdominal organ infection. Given that ascites is a good culture medium for bacteria, patients with cirrhosis are prone to SBP after ascites. SBP is a serious complication of advanced liver disease and chronic severe hepatitis, with a prevalence of 10–30% among hospitalized patients (10, 13). Different investigations reported varying hospital mortality rates in hospitalized patients with cirrhosis and SBP (20–40%) (14–17). SBP worsens the outcomes of chronic liver diseases and increases the risk of complications, such as renal and hepatic failure and portal hypertension (18, 19). SBP diagnosis follows the European Association for the Study of the Liver and the American Association for the Study of Liver Diseases guidelines, which indicate that ascites polymorphonuclear leukocyte counts combined with no intra-abdominal source of infection are suggestive of SBP (20, 21). However, ascites puncture is an invasive procedure, in which patient compliance is poor and the examination is time-consuming (24–48 h). The positive rate is as low as 35–65% (22). In some special cases, the patient's physical condition cannot carry out ascites puncture. On the basis of the current diagnostic strategies, many SBP cases can be misdiagnosed, leading to a delay in treatment or antibiotic abuse. Moreover, early clinical diagnosis of SBP is difficult because of its atypical clinical manifestations, few symptoms, or common symptoms, and signs of other diseases.

Although many studies have discussed the morbidity, diagnosis, and treatment of SBP, its early diagnosis still presents some difficulties. Currently, many risk factors and diagnostic studies of DC based on clinically recognized risk factors are small-sample studies. The model has not been further verified, and the availability and accuracy of the model in clinical use cannot be guaranteed. The clinical factors involved in the study are non-clinical common factors, in daily monitoring, doctors or nurses cannot be reminded of the risk of early detection of SBP. The study design has few clinical factors, strong intervenability and is easily affected by a single influencing factor. This study aimed to establish a simple and effective method for diagnosing whether SBP occurs in patients with DC based on large samples and common clinical indicators of DC. In this study, a nomogram was employed. In addition, we developed an online application based on the nomogram to diagnose SBP in patients with DC. This work can be used to clinically evaluate patients with DC to assess the risk of SBP.

## Materials and Methods

### Study Subjects

This study is a retrospective review. We collected data from 4,607 patients diagnosed with DC in A hospital (3,612 cases) and B hospital (770 cases) in the Big Data Platform of Medical Data Research Institute of Chongqing Medical University between July 2015 and December 2019.

The inclusion criterion was a diagnosis of DC between July 2015 and December 2019. Exclusion criteria were as follows: patients younger than 18 years old; patients suffering from acquired immunodeficiency syndrome; patients suffering from tumors; patients with secondary peritonitis; patients with tuberculous peritonitis; and indicators with a missing rate >30%.

The diagnosis of DC is based on LC, with complications of portal hypertension and (or) liver dysfunction. There is a basis for the diagnosis of LC: the diagnosis of LC is confirmed by liver biopsy, clinical, biochemical, and imaging data or past medical records, and the diagnosis is in accordance with the “Chinese guidelines on the management of liver cirrhosis” (23). The portal hypertension-related complications and symptoms include ascites, bleeding from esophageal gastric varices, sepsis, hepatic encephalopathy, and hepatorenal syndrome. The diagnostic criteria of SBP are based on the 2010 “European Society of Liver Diseases Cirrhosis and Ascites, SBP Guidelines” (24), which involve any two of the following indicators: clinical manifestations of fever, abdominal pain, abdominal tenderness, rebound pain, and other abdominal cavity symptoms and signs of internal infection; routine examination of ascites for white blood cells >0.5 × 10^9^/L and neutrophils >0.25 × 10^9^/L; and positive bacterial culture of ascites.

### Demographic Features and Clinical Characteristics

This study included common clinical indicators of patients with LC by consulting clinical experts and inquiring about the related literature. Etiology of LC: hepatitis B, hepatitis C, alcoholic liver, biliary hepatitis, and autoimmune hepatitis. Common complications of patients with SBP: upper gastrointestinal bleeding and hepatic encephalopathy. Laboratory data: blood routine, liver function, renal function, electrolytes, blood coagulation function indexes, and model foe end stage liver disease (MELD). Basic information and medical history information: age, sex, smoking history, drinking history, high blood pressure, and diabetes. C-reactive protein (CRP) >8 mg/L is defined as abnormal CRP, whereas procalcitonin >0.5 μg/L is defined as abnormal procalcitonin.

### Statistical Analysis

We collected 3,873 patients with DC, including 1,286 female patients and 2,587 male patients. In the first step, we used SPSS24.0 to perform single-factor analysis on the SBP group and non-SBP group, and the test standard was set to *p* < 0.05. The enumeration data were expressed by rate and percentage, and the chi-square test was used for comparison between groups. All the quantitative variables do not obey the normal distribution. Those were represented by median and interquartile range (IQR) [M(Q25–Q75)], and comparison between groups was represented by the Mann–Whitney *U*-test. We use Originlab to draw stacked histograms of antibiotic usage. In the second step, with the aid of R software (version 3.6.2; https://www.R-project.org), we randomly divided the patients into two groups, namely, traindata (*n* = 2,685) and internal validation set (*n* = 1,152), according to a theoretical ratio of 7:3. In the third step, we adopted the least absolute shrinkage and selection operator (LASSO) regression method to analyze variables. LASSO was applied for data dimensionality reduction. The LASSO regression model employs a double-standard error by constructing a penalty function. Concerning the characteristics of this method, we screened suitable and effective risk factors for patients with DC and SBP in LASSO regression analysis and selected nine characteristic factors. Variables selected through logistic regression analysis were considered for odds ratio (*OR*) and *p* with 95% *CI*, and the statistical significance levels were all two-sided. On the basis of the logistic regression results, we selected risk factors with *p* < 0.05 and constructed a nomogram prediction model. In this study, seven variables were selected. For the validation of the model, we calculated C index, receiver operating characteristic (ROC) curve, and dynamic component analysis (DCA) (22).

## Result

### Baseline Characteristics

A total of 3,873 patients were included for analyses. The baseline data of enrolled patients are summarized in Table 1. In patients with DC, the prevalence of SBP was 20.75%. The total cohort comprised 2,587 (67.42%) men, and the proportion of men in patients with SBP was high, i.e., 599 men (75.25%). Among all enrolled patients with DC, the median age was 53 years. In terms of suffering from underlying diseases, the proportions of hypertension and diabetes were 12.95 and 19.36%, respectively. This study included five common complications associated with DC, and the incidence of the five common complications is as follow: esophageal and gastric varices without bleeding (36.33%), upper gastrointestinal bleeding (31.56%), hypersplenism (21.53%), hypoproteinemia (11.78%), and hepatic encephalopathy (9.41%). Hepatitis B was the leading etiology of LC (56.50%). The rates of smoking and drinking were 47.20 and 47.15%, respectively.

**Table 1:** Characteristics of the participants in the SBP group and non-SBP group.

**Variable**	**Total (*n* = 3,837)**	**SBP (*n* = 796)**	**Non-SBP** **(*n* = 3,041)**	**Z/χ^2^**	***P***
Sex (Male)	2,587 (67.42%)	599 (75.25%)	1,988 (65.37%)	28.026	<0.001
Hypertension (Yes)	497 (12.95%)	80 (10.05%)	417 (13.71%)	7.505	0.006
Diabetes (Yes)	743 (19.36%)	134 (16.83%)	609 (20.03%)	4.117	0.042
HE (Yes)	361 (9.41%)	131 (16.46%)	230 (7.56%)	58.55	<0.001
UGIB (Yes)	1,211 (31.56%)	181 (22.74%)	1,030 (33.87%)	36.192	<0.001
Hypoproteinemia (Yes)	452 (11.78%)	138 (17.34%)	314 (10.33%)	29.84	<0.001
Hypersplenism (Yes)	826 (21.53%)	138 (17.34%)	688 (22.62%)	10.441	0.001
EGVWB (Yes)	1,394 (36.33%)	198 (24.87%)	1,196 (39.33%)	56.985	<0.001
Hepatitis B	2,168 (56.50%)	510 (64.07%)	1,658 (54.52%)	23.405	<0.001
Alcoholic liver	837 (21.81%)	216 (27.14%)	621 (20.42%)	16.678	<0.001
Biliary hepatitis	362 (9.43%)	54 (6.78%)	308 (10.13%)	8.258	0.004
Hepatitis C	271 (7.06%)	40 (5.03%)	231 (7.60%)	6.353	0.012
Autoimmune hepatitis	279 (7.27%)	38 (4.77%)	241 (7.93%)	9.291	0.002
Smoking (Yes)	1,811 (47.20%)	438 (55.03%)	1,373 (45.15%)	24.688	<0.001
Drinking (Yes)	1,809 (47.15%)	430 (54.02%)	1,379 (45.35%)	19.045	<0.001
PCT (Abnormal)	729 (19.00%)	355 (44.60%)	374 (12.30%)	427.663	<0.001
CRP (Abnormal)	1,376 (35.86%)	452 (56.78%)	924 (30.38%)	191.149	<0.001
Age (Year)	53.00 (46.00, 63.00)	52.00 (45.00, 62.00)	54.00 (46.00,6 3.00)	−3.799	<0.001
HD (Days)	10.00 (6.00, 17.00)	15.00 (9.00, 22.00)	9.00 (6.00, 15.00)	−13.404	<0.001
NE (%)	68.00 (59.00, 76.40)	74.00 (66.20, 81.91)	66.30 (57.40, 74.50)	−15.564	<0.001
NE (×109/L)	2.72 (1.79, 4.23)	3.94 (2.60, 6.53)	2.49 (1.66, 3.74)	−17.308	<0.001
MCV (fL)	91.50 (85.10, 97.00)	92.00 (85.33, 98.60)	91.30 (85.00, 96.60)	−3.141	0.002
MCH (pg)	31.00 (27.90, 33.10)	31.90 (29.43, 34.00)	30.70 (27.60, 32.90)	−8.412	<0.001
MCHC (g/L)	336.00 (321.00, 347.00)	343.50 (329.00, 356.00)	333.00 (319.00, 345.00)	−12.884	<0.001
LY% (%)	21.00 (14.22,28.80)	15.46 (10.30,21.40)	22.60 (15.70,30.10)	−16.953	<0.001
LY (×109/L)	0.85 (0.57,1.25)	0.83 (0.56,1.26)	0.85 (0.58,1.25)	−0.922	0.357
WBC (×109/L)	4.22 (3.00, 6.10)	5.49 (3.86, 8.59)	3.99 (2.85, 5.53)	−14.735	<0.001
Red blood cell count (×10^9^/L)	3.49 (2.88,4.04)	3.38 (2.78,3.93)	3.51 (2.92,4.07)	−3.92	<0.001
PLT (×109/L)	70.00 (46.00, 110.75)	73.00 (47.00, 117.75)	69.00 (46.00, 109.00)	−1.691	0.091
Hb (g/L)	104.00 (84.00, 123.00)	104.00 (84.00,123.00)	103.00 (84.00, 123.00)	−0.119	0.906
Calcium (mmol/L)	2.10 (1.99, 2.21)	2.04 (1.95, 2.16)	2.12 (2.01, 2.22)	−9.994	<0.001
Phosphorus (mmol/L)	1.05 (0.90, 1.20)	1.03 (0.87, 1.18)	1.05 (0.91, 1.20)	−2.676	0.007
Magnesium (mmol/L)	0.78 (0.71, 0.85)	0.78 (0.70, 0.86)	0.78 (0.71, 0.85)	−0.741	0.459
Potassium (mmol/L)	3.89 (3.59, 4.21)	3.88 (3.54, 4.26)	3.89 (3.60, 4.20)	−0.091	0.928
Sodium (mmol/L)	139.00 (136.00, 141.40)	138.52 (135.30, 141.00)	139.10 (136.00, 141.50)	−3.486	<0.001
Chlorine (mmol/L)	102.80 (99.20, 105.70)	102.30 (98.30, 105.30)	103.00 (99.40, 105.80)	−3.665	<0.001
Albumin (g/L)	31.40 (28.30, 35.40)	31.20 (28.25, 35.20)	31.50 (28.30, 35.50)	−0.98	0.327
Total bilirubin(umol/L)	27.80 (17.00, 62.60)	70.90 (27.50, 231.35)	24.80 (15.70, 45.80)	−19.89	<0.001
Total bile acid(umol/L)	34.80 (13.63,86.50)	71.50 (26.75,161.70)	30.10 (12.00,69.10)	−15.566	<0.001
Urea(mmol/L)	5.30 (4.09, 7.19)	5.68 (4.14, 8.21)	5.21 (4.08, 7.03)	−3.864	<0.001
Uric acid(umol/L)	288.35 (213.73, 375.08)	263.85 (170.95, 370.70)	293.55 (224.33, 375.58)	−5.925	<0.001
Creatinine (umol/L)	67.00 (55.50, 84.50)	67.00 (54.85, 86.78)	67.00 (55.60, 83.90)	−0.288	0.773
ALP (IU/L)	115.00 (83.00, 161.00)	124.00 (90.00, 167.50)	112.00 (81.00, 158.00)	−4.28	<0.001
GGT (IU/L)	52.00 (28.00, 111.00)	60.00 (31.00, 114.00)	50.00 (27.00, 110.00)	−3.275	0.001
ALT	30.00 (19.00, 54.75)	39.00 (22.00, 118.00)	28.00 (19.00, 48.00)	−9.899	<0.001
PAB (mg/L)	80.00 (51.00, 123.00)	52.00 (34.50, 78.00)	88.00 (58.00, 132.00)	−20.046	<0.001
PT(S)	16.70 (15.10, 19.50)	19.60 (16.73, 24.40)	16.30 (14.80, 18.50)	−19.734	<0.001
INR	1.36 (1.19, 1.65)	1.67 (1.36, 2.21)	1.31 (1.16, 1.54)	−19.783	<0.001
MELD	11.27 (8.02, 16.52)	17.24 (10.73, 24.06)	10.49 (7.57, 14.59)	−18.903	<0.001
Total protein(g/L)	66.10 (59.53, 72.80)	62.60 (56.70, 68.30)	67.10 (60.40, 73.70)	−11.287	<0.001

*HE, hepatic encephalopathy; UGIB, upper gastrointestinal bleeding; EGVWB, esophageal and gastric varices without bleeding; PCT, Procalcitonin; CRP, C-reactive protein; HD, hospitalization days; NE%, percentage of neutrophils; NE, the absolute number of neutrophils; MCV, mean corpuscular volume; MCH, mean corpuscular volume; MCHC, mean red blood cell hemoglobin concentration; LY%, lymphocyte percentage; LY, lymphocyte count; WBC, white blood cell count; PLT, platelet count; Hb, hemoglobin concentration; ALP, alkaline phosphatase; GGT, γ-glutamyl transpeptidase; ALT, alanine aminotransferase; PAB, prealbumin; PT, prothrombin time; INR, international normalized ratio; MELD, a model for end-stage liver disease*.

The percentages of abnormal CRP level and procalcitonin in patients with SBP were 56.78 and 30.38%, respectively, or 44.60 and 12.30%, respectively, compared with those without SBP. The median hospitalization time was 15 days (9, 22) in patients with SBP and 9 days (6, 15) in those without SBP. The median total bilirubin content was 70.90 μmol/L (27.50, 231.35) in patients with SBP and 24.80 μmol/L (15.70, 45.80) in those without SBP. The median prothrombin time and international normalized ratio levels of patients with DC and SBP were 19.60 s (16.73, 24.40) and 1.67 (1.36, 2.21), respectively, whereas those of patients without SBP were 16.30 s (14.80, 18.50) and 1.31(1.16, 1.54). The median MELD level of patients with DC and SBP was 17.24 (10.73, 24.06), whereas that of patients without SBP was 10.49 (7.57, 14.59). Meanwhile, we listed the use of antibiotics in the SBP and non-SBP groups (Supplementary Tables 1, 2 and Supplementary Figures 1, 2 for details).

### Univariate Analysis and LASSO Regression

A total of 50 indicators were included in this study, and seven indicators were not statistically significant after univariate analysis. For example, no statistical significance was found between patients with SBP and patients without SBP in lymphocyte count, platelet count, hemoglobin concentration, magnesium ion concentration, potassium ion concentration, albumin concentration, and creatinine concentration (*p* > 0.05). The remaining variables were statistically significant, such as CRP level, procalcitonin level, length of hospital stay, calcium concentration, prothrombin time, and MELD score (*p* < 0.05). The analysis results are shown in Table 1.

**Figure 1:**
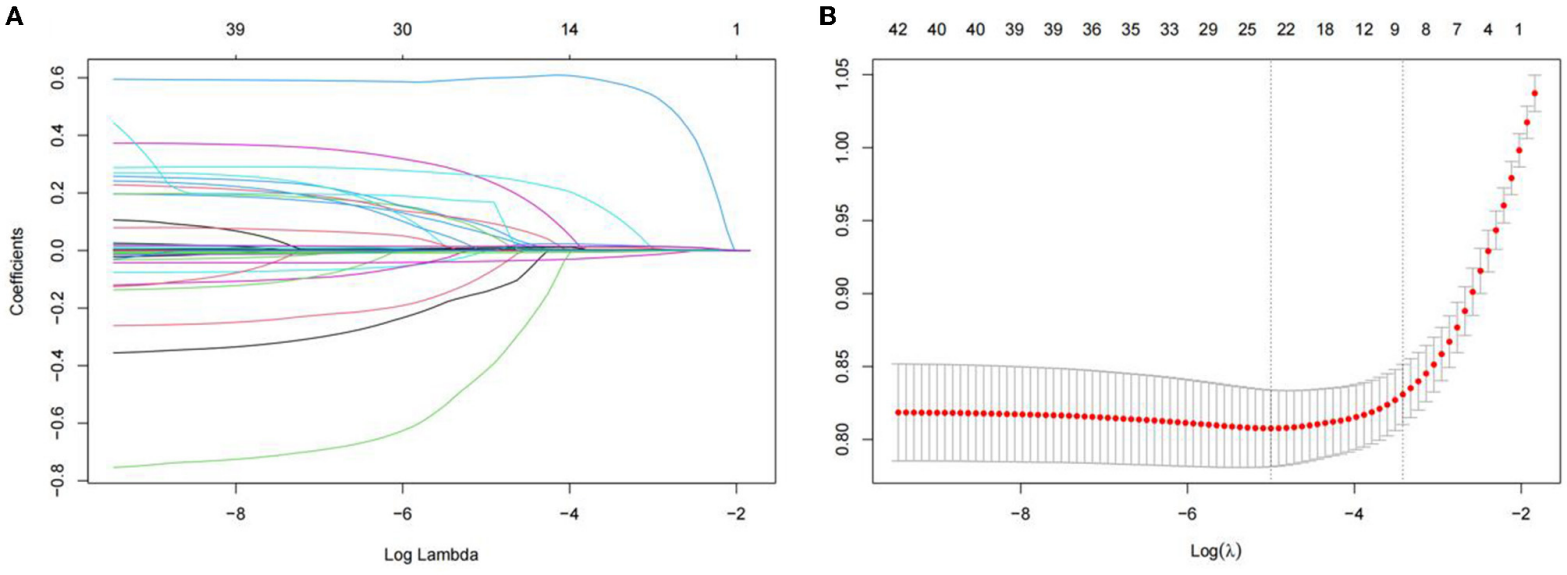
Demographic and clinical feature selection using the LASSO binary logistic regression model. **(A)** The selection of the best parameter (lambda) in the LASSO model uses five-fold cross-validation with the lowest standard. The relationship curve between partial likelihood deviation (binomial deviation) and log(lambda)was plotted. Dotted vertical lines were drawn at the optimal values by using the minimum criteria and the 1 SE of the minimum criteria (the 1-SE criteria). **(B)** LASSO coefficient profiles of the 11 features. A coefficient profile plot was produced against the log (lambda) sequence. Vertical line was drawn at the value selected using five-foldcross-validation. LASSO, least absolute shrinkage and selection operator; SE, standard error.

The least absolute shrinkage and selection operator regression analysis requires consistent initial estimation of regression coefficients, which can be used to reduce the dimension of complex variables and improve the accuracy of the model (25). In this study, LASSO regression analysis was conducted to select nine predictors from the statistically significant univariate variables (Figures 1A,B), thereby consolidating our model. The nine predictors were CRP level, absolute neutrophil count, mean red blood cell hemoglobin concentration, lymphocyte percentage, total bilirubin, prealbumin, prothrombin time, procalcitonin level, and MELD (Table 2).

**Table 2:** Least absolute shrinkage and selection operator (LASSP) regression coefficient table.

**Variable**	**Coefficients**	**Lambda.1se**
Procalcitonin level	0.580	0.033
C-reactive protein level	0.113	
Absolute number of neutrophils	0.011	
Mean red blood cell hemoglobin concentration	0.001	
Lymphocyte percentage	−0.022	
Total bilirubin	0.003	
Prealbumin	−0.005	
Prothrombin time	0.018	
MELD	0.014	

### Development of a Nomogram

The nine variables of LASSO regression were brought into a multivariate logistic regression, and stepwise logistic regression based on the AIC criterion was performed by step() function. The values of *p* of seven risk characteristic factors were proven to be significant in the model (Table 3).

**Table 3:** Results of multivariate logistic regression model.

**Variable**	**β**	**SE**	**OR (95%CI)**	***P***
Procalcitonin level	0.680	0.132	1.975 (1.522,2.556)	0.000
C-reactive protein level	0.333	0.118	1.395 (1.107,1.755)	0.005
MCHC	0.010	0.003	1.010 (1.004,1.016)	0.001
Lymphocyte percentage	−0.046	0.006	0.955 (0.943,0.967)	0.000
Total bilirubin	0.003	0.001	1.003 (1.002,1.004)	0.000
Prealbumin	−0.010	0.002	0.990 (0.987,0.993)	0.000
Prothrombin time	0.037	0.012	1.038 (1.015,1.063)	0.002

**Figure 2:**
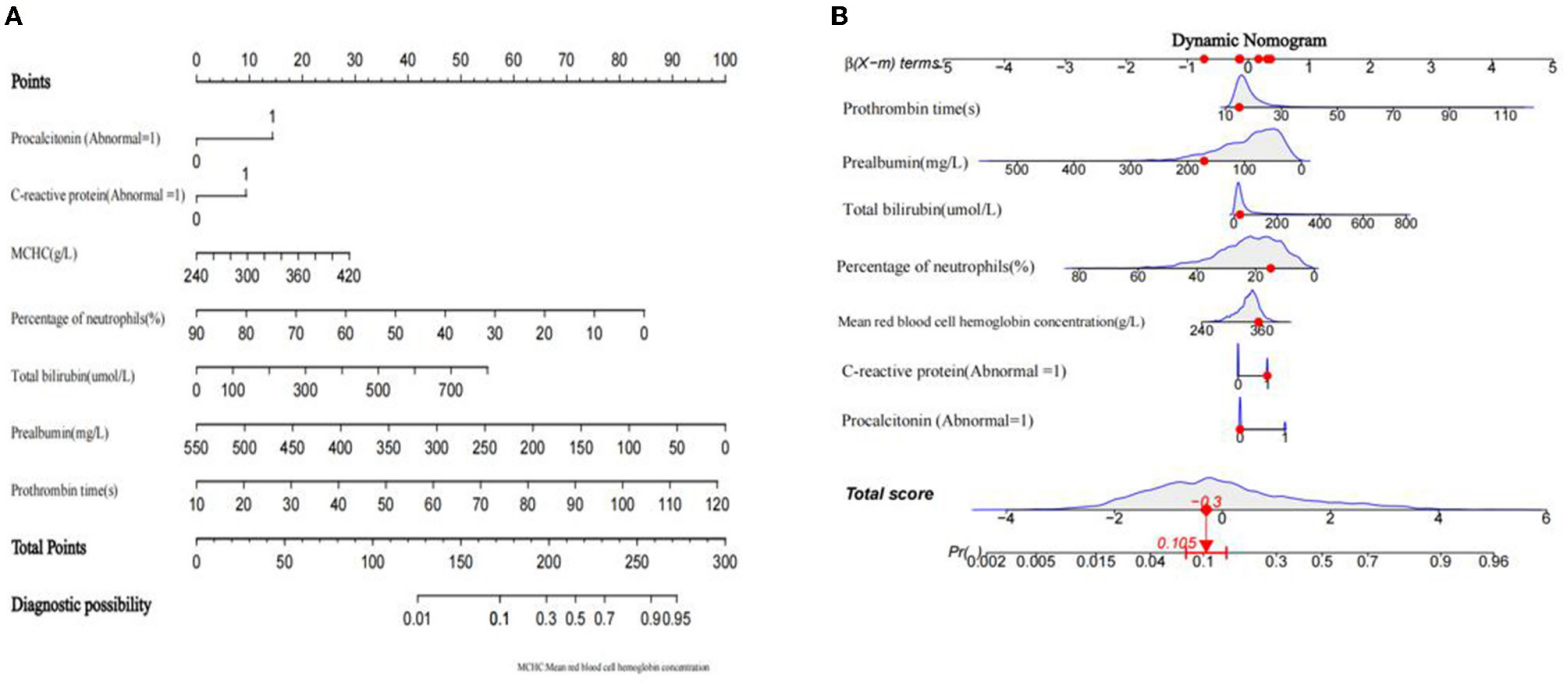
Developed a nomogram of decompensated cirrhosis combined spontaneous bacterial peritonitis model. **(A)** spontaneous bacterial peritonitis nomogram for patients with decompensated cirrhosis was developed in the traindata, with prothrombin time, prealbumin, total bilirubin, lymphocyte ratio, mean red blood cell hemoglobin concentration, procalcitonin level and C-reactive protein level incorporated. **(B)** An example of nomogram.

The diagnostic nomogram that integrated seven selected features (*p* < 0.05) for the incidence of SBP in the training cohort is shown in Figure 2A. The values of each risk factor were assigned a score on the point scale axis. By adding each single score and using that value in the total point scale axis, the total score could be easily calculated to the probability of SBP. For the convenience of clinicians, we have provided the nomogram as a web-based calculator tool (https://cqmuxss.shinyapps.io/dynnomappdc_and_sbp/). Doctors can enter the indicators for each patient to automatically calculate the patient's probability of SBP. To provide a plain and clear illustration of the integrated model, an example of a patient with DC is shown in Figure 2B. If the subject had an abnormal CRP level, a normal procalcitonin level, mean red blood cell hemoglobin concentration of 353 g/L, a lymphocyte percentage of 14.92%, a total bilirubin count of 27.1 μmol/L, a prealbumin count of 171 mg/L, and a prothrombin time of 14.9 s, then the probability of SBP was estimated to be 10.5%.

### Verification of the Nomogram

**Figure 3:**
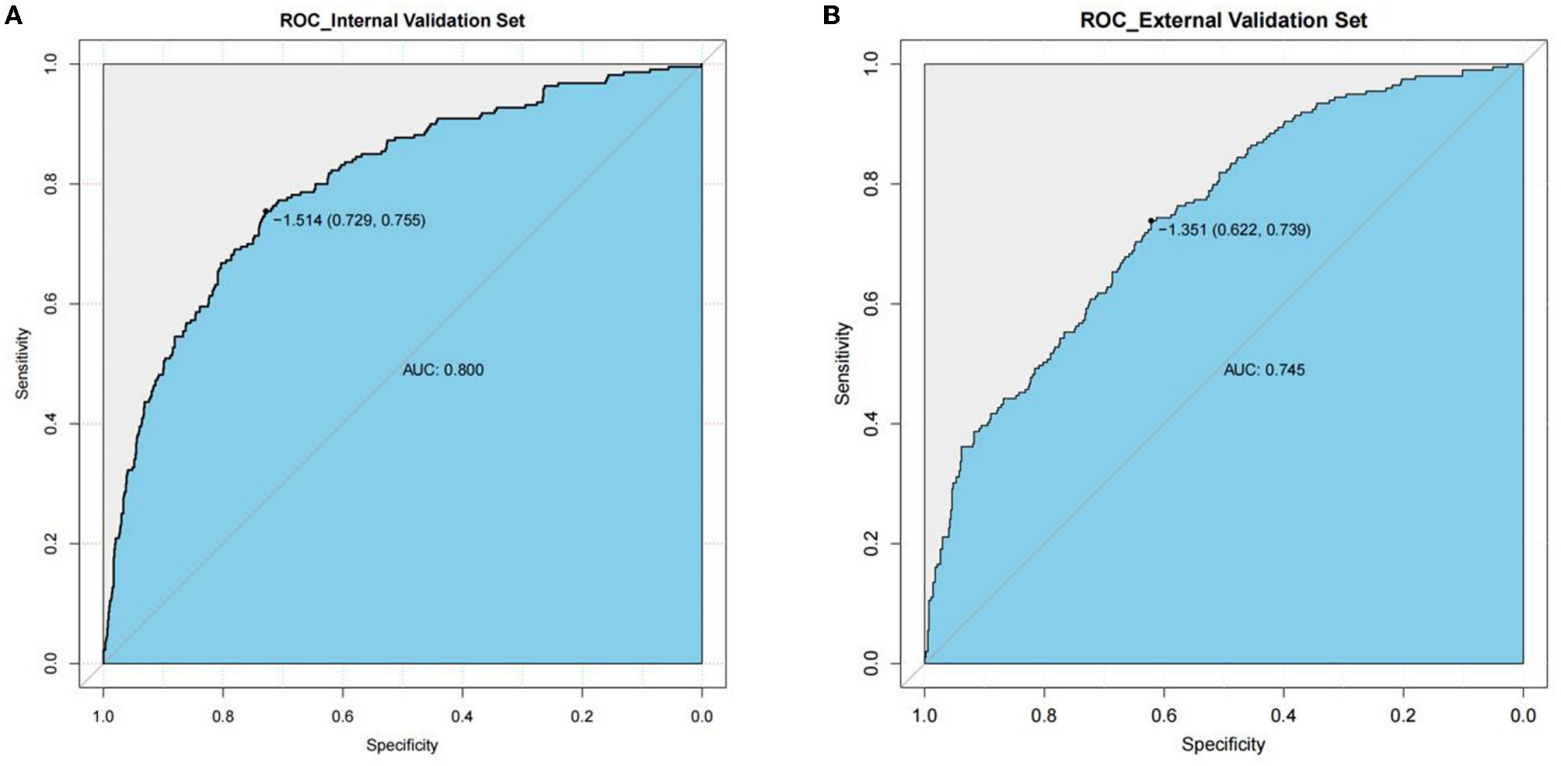
AUC of the ROC curve in internal validation set and external validation set. Internal validation set **(A)**, and external validation set **(B)**. The y axis means the true positive rate of the risk prediction of decompensated cirrhosis patients with spontaneous bacterial peritonitis. The x axis means the false positive rate of the risk prediction of decompensated cirrhosis patients with spontaneous bacterial peritonitis.

Good discrimination was obtained by validating the model using internal and external validation sets. In the internal verification set, the AUC was 0.800 and the best cutoff point for the model was −1.514, where the specificity was 72.9% and the sensitivity was 75.5% (Figure 3A). In the external verification set, the AUC was 0.745 and the best cutoff point for the model was −1.351, where the specificity was 62.2% and the sensitivity was 73.9% (Figure 3B).

**Figure 4:**
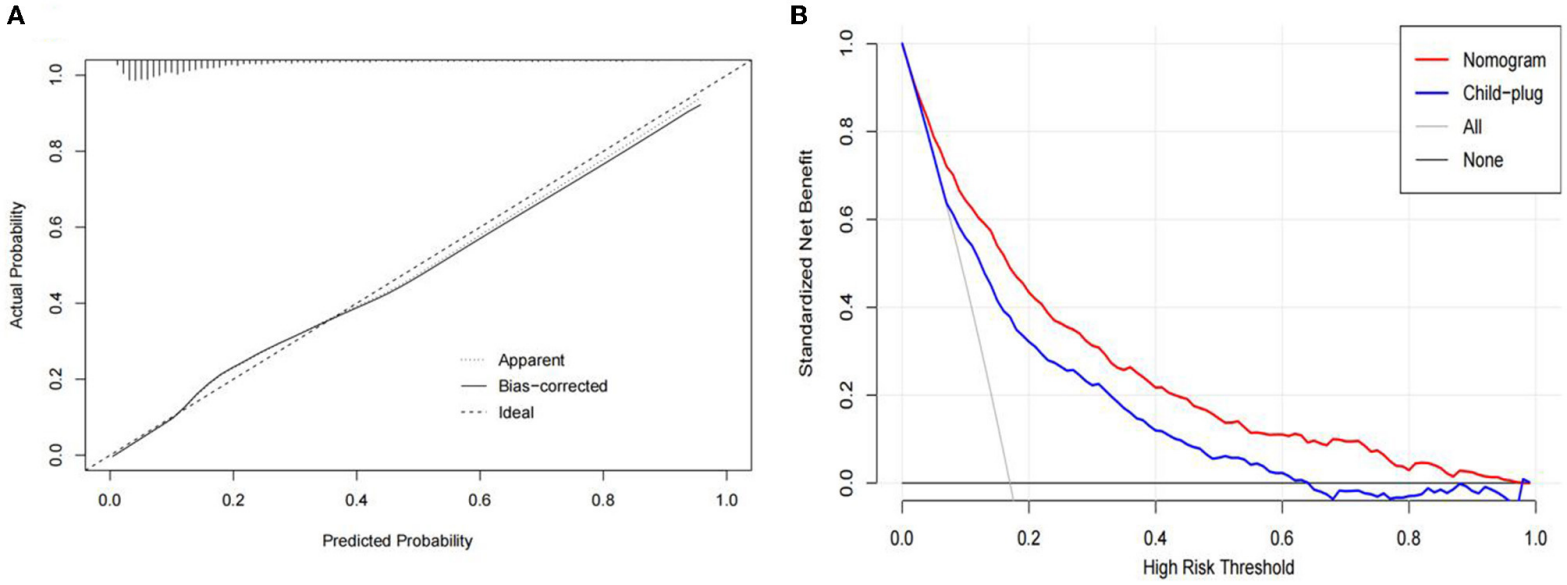
**(A)** Calibration curves of the spontaneous bacterial peritonitis incidence risk nomogram prediction in the traindata. The diagonal line represents the reference line of complete coincidence between the predicted value and the actual value, The apparent dotted line represents the actual situation of the prediction model, and the bias-corrected solid line represents the actual situation of the prediction model after correction. **(B)** Decision curve analysis of the nomogram and child-plug. The y-axis represents standardized net benefit.

In this study, the bootstrap resampling method was used to calibrate the calibration curve of the logistic model (Figure 4A). The diagonal line represents the reference line where the predicted value and the actual value are completely coincident, and the apparent dotted line represents the actual situation of the prediction model. The bias-corrected solid line represents the actual situation of the corrected prediction model. The C-index and Brier score were 0.830 (95% *CI*: 0.811–0.849) and 0.117 (95% *CI*: 0.109–0.126), respectively. We compared the nomogram and Child-plug based on DCA. DCA visually showed that the nomogram had a superior overall net benefit within the wide and practical ranges of threshold probabilities. It further indicated that our nomogram conferred significantly high clinical net benefit, which is of great value for the accurate individualized assessment of the incidence of SBP (Figure 4B). These values suggested that the diagnostic model demonstrated good performance.

## Discussion

Spontaneous bacterial peritonitis is a serious threat to the quality of life and survival time of patients with DC. Early detection of the risk of SBP in patients with DC is helpful to improve the quality of life of patients. The survival time of patients can be prolonged, and antibiotics can be better managed and utilized. In this study, we found that mean red blood cell hemoglobin concentration (*OR* = 1.010, 95% *CI*: 1.004–1.016), prothrombin time (*OR* = 1.038, 95% *CI*: 1.0151.063), lymphocyte percentage (*OR* = 0.955, 95% *CI*: 0.943–0.967), prealbumin (*OR* = 0.990, 95% *CI*: 0.987–0.993), total bilirubin (*OR* = 1.003 95% *CI*: 1.002–1.004), abnormal CRP level (*OR* = 1.395, 95% *CI*: 1.107–1.755), and abnormal procalcitonin levels (*OR* = 1.975, 95% *CI*: 1.522–2.556) were associated with a higher incidence of SBP. Furthermore, we developed an effective prognostic nomogram composed of seven features. The nomogram had significantly high sensitivity and specificity to distinguish individuals with SBP from DC. DCA further indicated that our nomogram conferred significantly high clinical net benefit, which is of great value for the accurate individualized assessment of the occurrence of SBP.

Several studies have shown that patients with SBP have higher CRP levels than patients without SBP (26–29). In this study, patients with SBP and abnormal CRP levels accounted for 56.78%, which was significantly higher than patients with non-SBP 30.38%, consistent with previous studies. After trauma and infection, CRP increased significantly, and its application in disease diagnosis can assist in judging whether patients have an inflammatory reaction and bacterial infection. After SBP in patients with DC, patients usually have an acute inflammatory response. The level of CRP significantly increases. In addition, studies have shown that elevated CRP levels are helpful in diagnosing SBP (30–32). Procalcitonin is a precursor of calcitonin that is normally secreted by the C cells of the thyroid gland in healthy individuals. In the absence of infection, extra-thyroid expression of the procalcitonin gene is suppressed in liver, lung, kidney, adrenal tissue, monocytes, granulocytes, testis, prostate, and small intestine (33). Bacterial infection induces a general increase in procalcitonin gene expression, resulting in the constitutive release of procalcitonin from parenchymal tissue throughout the body. Procalcitonin is significantly elevated during the systemic response to infection, and this effect can be detected 2-4 h after the inflammatory response; it is known as a new inflammatory biomarker of bacterial infection (34–36). Su DH, Connert S, Cekin Y, et al. have shown that procalcitonin is a marker of inflammation for the diagnosis of SBP and can be used by clinicians as an aid in the rapid and accurate diagnosis of SBP (37–39).

The liver can synthesize all coagulation factors and fibrinolytic factors except tissue factors and calcium ions. The coagulation factor is the most sensitive indicator to evaluate the status of liver function. Measuring the level of coagulation factor can quickly reflect the status of liver function. Prolonged prothrombin time indicates decreased liver function, and SBP should be considered when prolonged prothrombin time occurs. Many scholars have reported that total bilirubin is an independent risk factor for cirrhosis patients complicated with SBP (40–42). The increase in total bilirubin reflects the situation of liver damage and the decline of liver synthetic function. Total bilirubinostasis can damage the function of Kupffer cells and aggravate liver function damage; it is accompanied by a significant decline in the function of the monocyte-macrophage system. It can easily lead to bacterial migration and bacteremia, which can increase the possibility of SBP. The occurrence of SBP further increases the burden on the liver. Given their interaction, patients with cirrhosis with significantly increased total bilirubin levels need to be highly alert to the occurrence of SBP.

Prealbumin is an acute-phase protein with a relative molecular weight of 55 kDa. It participates in immune regulation and plays a role in maintaining the acid-base balance. Li Wq (3) pointed out that the distribution rate of serum albumin from intravascular to extravascular increases significantly during an infection, and the decomposition rate of albumin also increases significantly. Under physiological conditions, prealbumin is mainly present in serum and cerebrospinal fluid and has a shorter half-life (1.9 days) than albumin (43). The short-term increase and decrease are more obvious. The detection of serum prealbumin level has important clinical value in the diagnosis, treatment, and prognosis of various liver diseases, such as DC(44). Early diagnosis has higher sensitivity in predicting the occurrence of SBP in patients with cirrhosis. We found that lymphocyte percentage and mean corpuscular hemoglobin concentration were not discussed in previous study of influencing factors, hoping to provide a conjecture for related researchers.

In this study, the predictors included in the SBP diagnostic model for patients with DC were common clinical factors that could be collected in patients with DC. Thus, the model is useful and applicable in daily practice. Several predictors involved included liver functional indicators and inflammatory indicators, which did not have a major influence on the diagnosis when a single factor was affected. The performance of the model was relatively stable. Compared with previous studies, the online nomograms we provided could help doctors make a quick diagnosis, help patients perform self-screening and provide the possibility of timely diagnosis and treatment of diseases in places where medical technology is backward. The model performs well in internal and external verification, making it applicable to a large number of patient groups.

Our study had some limitations. First, selection bias may exist due to the retrospective nature of the investigation. However, we used a multicenter and relatively large training cohort to build models, which was further subjected to spatial verification. Second, data on social support, level of education, and socioeconomic status were not available. Further research is warranted to explore the impact of these important indicators.

## Conclusion

This study developed and validated a diagnostic model based on common clinical features of patients with DC. This study is helpful for the early detection of patients with SBP, timely provision of reasonable symptomatic treatment, and improvement of quality of life and prognosis of patients with DC. The online nomogram is convenient for clinicians to use and can increase the acceptability of the model.

## Data Availability Statement

The data analyzed in this study cannot be disclosed due to the privacy of some patients. Requests to access these datasets should be directed to Shoushu Xiang, 718998832@qq.com.

## Ethics Statement

The studies involving human participants were reviewed and approved by ChongQing Medical University. Written informed consent for participation was not required for this study in accordance with the national legislation and the institutional requirements.

## Author Contributions

SX, JT, and WZ were involved in concept and design. SX, JT, TW, and CY drafted the manuscript. SX and CT performed the statistical analysis. WZ provided administrative, technical, and material support, had full access to all the data in the study, and take responsibility for the integrity of the data and the accuracy of the data analysis and supervision. YW was responsible for clinical consultation. All the authors critically revised the manuscript for important intellectual content, acquisition, analysis, or interpretation of data, and read and approved the final manuscript.

## Funding

This research was funded by the Intelligence Medicine Project of Chongqing Medical University (Grant no. ZHYX2019001).

## Conflict of Interest

The authors declare that the research was conducted in the absence of any commercial or financial relationships that could be construed as a potential conflict of interest.

## Publisher's Note

All claims expressed in this article are solely those of the authors and do not necessarily represent those of their affiliated organizations, or those of the publisher, the editors and the reviewers. Any product that may be evaluated in this article, or claim that may be made by its manufacturer, is not guaranteed or endorsed by the publisher.
