# Supplementary material for: Establishment and Validation of a Non-Invasive Diagnostic Nomogram to Identify Spontaneous Bacterial Peritonitis in Patients With Decompensated Cirrhosis
Source: Front Med (Lausanne). 2022 Jan 31;8:797363. doi: 10.3389/fmed.2021.797363 (PMC8842661; doi:10.3389/fmed.2021.797363)
Supplement: Supplementary file 1 [file Data_Sheet_1.docx]

**TABLE 1 Statistics on the use of various antibiotics**

| **Antibiotic category** | **SBP(n=796)** | **NOSBP(n=3041)** |
| --- | --- | --- |
| Semi synthetic penicillin | 411(51.63%) | 700(23.02%) |
| Carbapenem | 299(37.56%) | 474(15.59%) |
| Penicillins | 269(33.79%) | 468(15.39%) |
| Quinolones | 230(28.89%) | 350(11.51%) |
| Cephamycin | 131(16.46%) | 247(8.12%) |
| Third-generation cephalosporin | 44(5.53%) | 59(1.94%) |
| Triazoles | 39(4.90%) | 41(1.35%) |
| First-generation cephalosporin | 41(5.15%) | 33(1.09%) |
| Nitroimidazoles | 24(3.02%) | 45(1.48%) |
| Others | 84(10.55%) | 117(3.85%) |


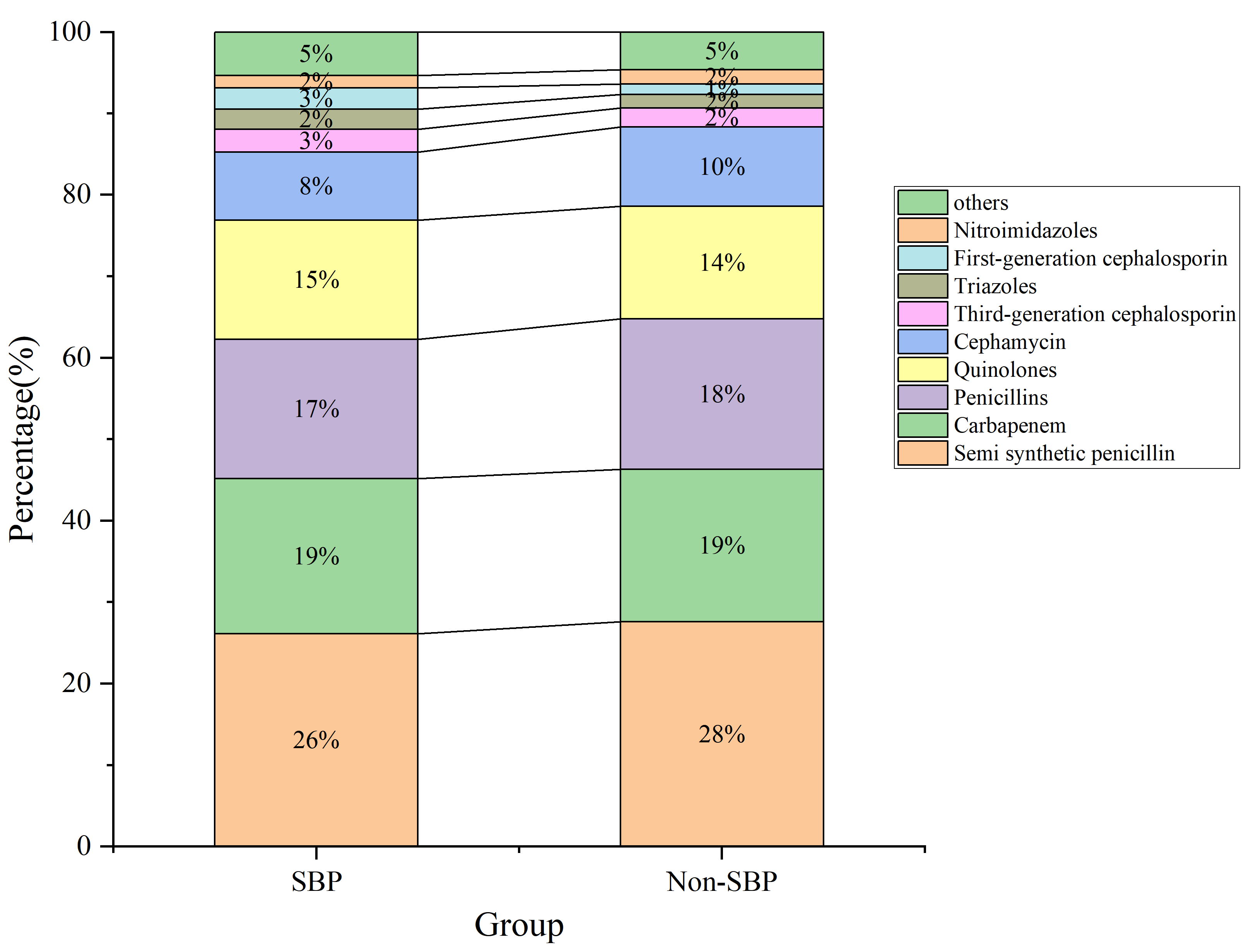


**FIGURE 1 Percentage distribution of various antibiotics**

**TABLE 2 Types of antibiotics used by a single patient**

| Number of species | Number of patients (n=3837) | SBP(n=796) | non-SBP(n=3041) |
| --- | --- | --- | --- |
| 1 | 1162(30.28%) | 333(41.83%) | 829(27.26%) |
| 2 | 618(16.11%) | 249(31.28%) | 369(12.13%) |
| 3 | 276(7.19%) | 116(14.57%) | 160(5.26%) |
| 4 | 122(3.18%) | 62(7.79%) | 60(1.97%) |
| >4 | 65(1.69%) | 24(3.02%) | 41(1.35%) |


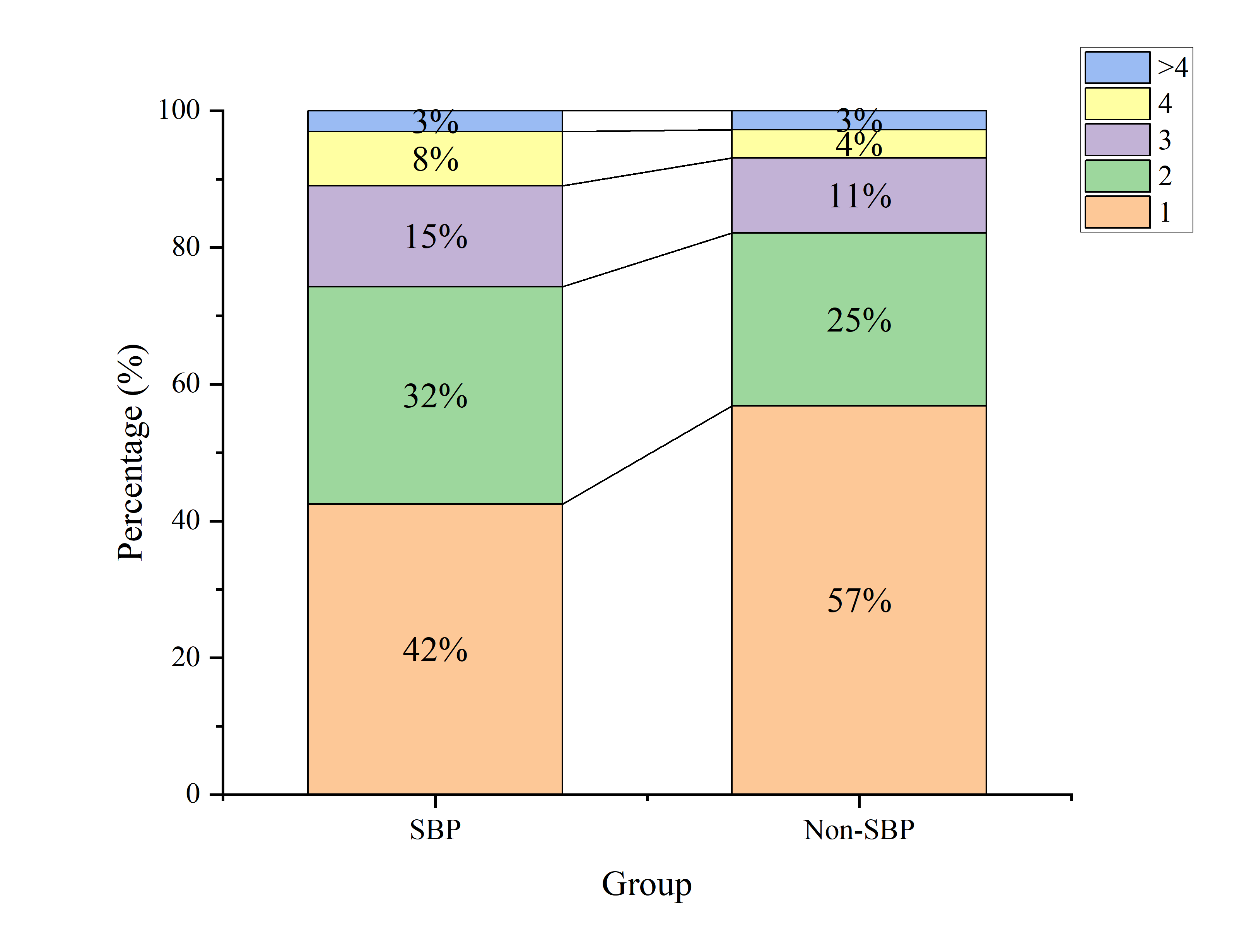


**FIGURE 2 Percentage distribution of types of antibiotics used by a single patient**
